# Lifestyle Behaviours and Plasma Vitamin C and **β**-Carotene Levels from the ELAN Population (Liège, Belgium)

**DOI:** 10.1155/2011/494370

**Published:** 2011-03-06

**Authors:** Joël Pincemail, Sophie Vanbelle, Fabien Degrune, Jean-Paul Cheramy-Bien, Corinne Charlier, Jean-Paul Chapelle, Didier Giet, George Collette, Adelin Albert, Jean-Olivier Defraigne

**Affiliations:** ^1^Department of Cardiovascular Surgery, University of Liège—CHU, B35 Sart Tilman, 4000 Liège, Belgium; ^2^Research Centre for Experimental Surgery (CREDEC), University of Liège—CHU, B35 Sart Tilman, 4000 Liège, Belgium; ^3^Department of Medical Informatics and Biostatistics, University of Liège—CHU, B35 Sart Tilman, 4000 Liège, Belgium; ^4^Department of Toxicology, University of Liège—CHU, B35 Sart Tilman, 4000 Liège, Belgium; ^5^Laboratory of Clinical Chemistry, University of Liège—CHU, B35 Sart Tilman, 4000 Liège, Belgium; ^6^Department of General Practice, University of Liège—CHU, B35 Sart Tilman, 4000 Liège, Belgium

## Abstract

Several factors, including fruit and vegetables intakes, have been shown to significantly influence the plasma concentrations of the two antioxidants vitamin C and *β*-carotene. Deficiency levels of 6 mg/L (34.2 *μ*M) for vitamin C and of 0.22 mg/L (0.4 *μ*M) for *β*-carotene have been suggested below which cardiovascular risk might be increased. The present study performed on 897 presumably healthy subjects aged 40–60 years aimed to examine how modifiable lifestyle factors may be related to vitamin C and/or *β*-carotene deficiency. Gender, smoking, lack of regular physical activity and of daily fruit consumption (≥2/day), and social status (in particular, unemployment) were found to be significant risk factors for vitamin C deficiency. For *β*-carotene deficiency, the same factors were identified except social status; moreover, overweight and OC use in women were also found to have a deleterious effect. For non exposed subjects, the probability of developing vitamin C deficiency was 4% in men and 2.4% in women. This probability increased to 66.3% for men and to 44.3% for women (and even to 50.4% under OC use), when all risk factors were present. For *β*-carotene deficiency, the corresponding probabilities were equal to 29.7% in men and 13.7% in women (no risk factor present), and to 86.1% for men and 69.9% (91.6% for OC use) for women (all factors present), respectively.

## 1. Introduction

Clinical and epidemiological studies have identified several factors that increase the risk of coronary heart disease and heart attack. These factors include gender, ageing, heredity, smoking, hypercholesterolemia, hypertension, physical inactivity, obesity and overweight, and diabetes mellitus. Risk factors often occur in clusters and may build on one another, such as obesity leading to diabetes and elevated blood pressure. When grouped together, as in the metabolic syndrome, these factors lead to an even greater risk of coronary artery disease. However, coronary artery disease may also possibly develop without any known risk factors. Special attention has been given to the effect of psychological stress, oral contraceptives, excessive alcohol intake, sleep apnea, elevated C-reactive protein levels, fibrinogen, homocysteine and lipoprotein and, finally the presence of an oxidative stress. Sies [1] has defined oxidative stress as an imbalance between oxidants (e.g., free radical species derived from oxygen) and antioxidants in favour of the oxidants, leading to a disruption of redox signalling and/or molecular damage. A large number of studies have indicated that oxidative damages to proteins, lipids or DNA resulting from an increased production of reactive oxygen species (ROS) from pathological processes or external origins are involved in the development of cardiovascular diseases and cancer [2]. 

To regulate ROS production and fight against their deleterious effect, the organism responds with a large and complex battery of antioxidants including enzymes, proteins, iron chelators, low molecular weight compounds, trace elements, and antioxidants arising from our diet; among them, vitamins C and E, carotenoids and polyphenols are particularly important. Many epidemiological and clinical studies have also shown that the lower the antioxidant status, the higher the risk of developing cardiovascular diseases and cancer [3–13]. A few prospective, large-scale, European studies have shown a negative correlation between vitamin C plasma levels and all-cause or cardiovascular mortality. Plasma vitamin C levels below the normal range 4–8.8 mg/L (or 23–50 *μ*M) were found to double the risk of developing cardiovascular disease and cancer [14–18]. For *β*-carotene, the upper reference limit was estimated to be 0.22 mg/L (or 0.4 *μ*M) [18]. 

Factors like male gender, age, race, body mass index (BMI), smoking, alcohol consumption, triacylglycerol concentration, and inadequate dietary antioxidant intakes contribute to lower plasma levels of antioxidants [19–28]. To our knowledge, so far no study did investigate how lifestyle behaviours such as smoking, physical activity, and diet alone or in association contribute to reach critical vitamin C (<6 mg/L or 34.2 *μ*M) and *β*-carotene (<0.22 mg/L or 0.4 *μ*M) levels. Currently, an increasing number of general practitioners strive to assess the antioxidant status of their patients for prevention purposes. In this respect, they need to be correctly informed about antioxidants critical levels from scientific studies. This prompted us to undertake a wide epidemiological study based on a sample of the Belgian population.

## 2. Material and Methods

### 2.1. Subjects

#### 2.1.1. Clinical Examination

ELAN (“Etude Liégeoise sur les ANtioxydants”) is a cross-sectional epidemiological study conducted from March through July 2006 as a joint project between the University of Liège, the University Hospital of Liège, and the public health services of the Province of Liège (Belgium). The Province of Liège, one of the 10 Belgian provinces, has 1.040.297 inhabitants for a geographic area of 3862 km^2^. A stratified random sample of 55 general practitioners working in the Province was selected as follows: 21 (38%) in urban environment, 15 (27%) in semiurban, and 19 (35%) in rural living environment. Each physician was asked to recruit in his/her practice 20 presumably healthy volunteers aged 40–60 years. Exclusion criteria included intake of antioxidant supplementation and previous history of cardiovascular diseases, diabetesm or cancer. A total of 897 eligible subjects were finally enrolled in the study: 349 (39%) men and 548 (61%) women. 

The day before the examination visit, subjects fasted for at least 12 h and were not allowed to drink fruit juice and to perform physical activity. The day of the visit, information including age, height, weight, consumption of fruits and vegetable by means of a home-made questionnaire, and intake of alcohol and oral contraceptive pills was collected. The Body Mass Index (BMI) was calculated from height and weight (kg/m^2^). Smoking (yes/no) and physical activity (inactive or active 2-3 times a week) were also recorded. Social status was classified in 7 categories, as follows: professionals (I), managerial and technical occupations (II), manual (IIIa) and nonmanual skilled workers (IIIb), partly skilled workers (IV), retired (Va), and unemployed (Vb). The study protocol was approved by the University Hospital Ethics Committee for medical research. All contacted volunteers received written information about the goal of the study and signed an informed consent form prior to enrolment.

#### 2.1.2. Analytical Procedures

Blood samples were drawn on EDTA or Na-heparin as anticoagulant. They were immediately centrifuged on site and plasma was frozen as aliquots on ice packs coming from a −80°C freezer and placed in a refrigerating box. For the assay of vitamin C [29], 0.5 mL plasma was immediately transferred to ice-cold tubes containing 0.5 mL of 10% metaphosphoric acid. The whole mixture was frozen on ice packs. Analyses were performed on the day of blood collection by a spectrophotometric method using the reduction of 2,6-dichlorophenolindophenol (Perkin Elmer Lambda 40 Norwalk, USA, sensitivity: 2 mg/L, inter- and intra-CV: 4 and 6%). Plasma *β*-carotene (sensitivity 0.022 mg/L, inter- and intra-CV: 5.35 and 10.73%) was determined by HPLC procedure (Alliance Waters, USA) coupled with a diode array detector (PDA 2996, Waters, USA) [30].

#### 2.1.3. References and Nonoptimal Values

Both vitamin C and *β*-carotene were analyzed in a routine way. Independently of gender, our reference values, as published earlier on a population of 128 healthy subjects, were 6.2–18.8 mg/L (35.3–107.1 *μ*M) for vitamin C and 0.05–0.62 mg/L (0.09–1.15 *μ*M) for *β*-carotene [31, 32]. They are in agreement with those published in the literature [20].

### 2.2. Statistical Analysis

Results were expressed as means ± standard deviation (SD) for quantitative variables, while frequencies and proportions (%) were used for categorical variables. Mean values between groups were compared by one-way analysis of variance or the Kruskal-Wallis nonparametric method if normality assumptions could not be fulfilled. Proportions were compared by the chi-squared test for contingency tables. Correlation coefficients (classical or Spearman) were calculated for measuring the association between two quantitative variables. Logistic regression analysis was used to predict vitamin C and *β*-carotene deficiency from risk factors. The association between deficiency and risk factors was measured by the odds ratio (OR) and its 95% confidence interval. Calculations were always carried out on the maximum number of data available. Missing data were not replaced. Results were considered to be significant at the 5% critical level (*P* < .05). Data analysis was carried out using SAS (version 9.1 for Windows) and S-PLUS (version 9.0) statistical packages.

## 3. Results

The gender-specific demographic, biometric, and clinical characteristics of the study population are given in Table 1. In the ELAN population, respectively 27% of women and 25% of men were smokers. These figures are in agreement with those found for the Belgian population [34]. Height, weight, BMI, and both systolic and diastolic blood pressures were significantly higher in men than in women. The dietary intake of vitamin C and *β*-carotene (mg/day) derived from fruits and vegetables consumption generally was significantly lower in men than in women (*P* < .0001). 

Table 2 displays vitamin C and *β*-carotene mean levels with respect to subject's characteristics given in Table 1. As expected, vitamin C and *β*-carotene values were lower by, respectively, 15 and 32% in men when compared to women (*P* < .0001). Smokers had significantly decreased vitamin C and *β*-carotene levels by 19% (*P* < .0001) and 39% (*P* < .0001) when compared to nonsmokers. By contrast, regular physical activity was associated with a mean increase of 9% for vitamin C (*P* < .0001) and 25% for *β*-carotene (*P* < .0001) when compared to sedentary lifestyle. A positive relationship was demonstrated between the amount of daily fruit intake and both vitamin C and *β*-carotene levels. The latter were, respectively, reduced by 27% and 46% (*P* < .0001) in non consumers when compared to people eating 2-3 fruits/day or more. The group apple-pear-grapes-banana, kiwi, citrus fruits, orange, and all kinds of red fruits and strawberry had a positive influence on vitamin C. In contrast, none of the vegetables except endives had a positive effect on vitamin C (data not shown). With respect to *β*-carotene, apricot, the group apple-pear-grapes-banana, kiwi, citrus, mango, and orange contributed to improve the plasma level of *β*-carotene but only asparagus, carrot, and tomato for vegetables (data not shown). For *β*-carotene only, a negative impact of overweight (BMI ≥ 25 kg/m²) was also observed (*P* < .0001). Women taking oral contraceptive had significantly lower *β*-carotene levels but vitamin C levels were unchanged. No relevant effect of blood pressure, intestinal disorders, environment, and age (< and >50 years) has been evidenced on the plasma level of both studied antioxidants. 

**Figure 1:**
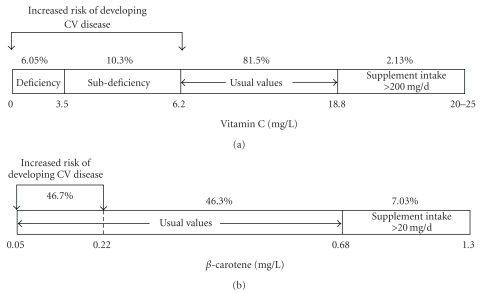
Plasma vitamin C and *β*-carotene distribution in the whole ELAN cohort (*n* = 897) with respect to usual values and the critical cutoff values of 6 mg/L (34.2 *μ*M) for vitamin C and 0.22 mg/L (0.4 *μ*M) for *β*-carotene. Usual (or reference) values were established on a population of 128 healthy volunteers and published earlier [31–33].

Figure 1 depicts the distribution of plasma vitamin C and *β*-carotene levels in men and women. Only 2.1% of the subjects had higher values than the upper reference value (18.8 mg/L). If a large majority (81.5%) of participants were within the recommended values in vitamin C (6.2–18.8 mg/L), it should be noted that 16.4% of them were, however, either in a subdeficiency (10.3%) or deficiency (6.1%) status with respect to the cutoff value of 6 mg/L (34.2 *μ*M). As far as the *β*-carotene is concerned, a small portion of the study subjects (7.0%) had higher concentration in *β*-carotene than the upper normal value (0.68 mg/L or 1.23 *μ*M) and more than 90% of the ELAN cohort had a plasma *β*-carotene concentration within the conventional and usual values (0.05–0.68 mg/L). It is worth noting that 46.7% of the studied population had plasma values below the critical point of 0.22 mg/L (0.4 *μ*M). 

By logistic regression analysis applied to vitamin C data (Table 3), it was found that male gender (OR = 1.70; *P* = .011), smoking (OR = 2.84; *P* < .0001), lack of physical activity (OR = 2.05; *P* = .0015), and intake of less than 2 fruits/day (OR = 2.96; *P* = .0003) were significant risk factors of vitamin C deficiency. Overweight (OR = 1.18; *P* = .42) and OC use for women (OR = 1.24; *P* = .61) were not significant. When adding the social status, the risk of vitamin C was significantly increased (OR = 1.34; *P* = .0044), when being unemployed compared to the other professional categories. For *β*-carotene deficiency, logistic regression analysis showed that male gender (OR = 2.67; *P* < .0001), smoking (OR = 3.03; *P* < .0001), overweight (OR = 2.28; *P* < .0001), lack of physical activity (OR = 1.51; *P* = .0091), intake of <2 fruits/day (OR = 1.41; *P* = .045), and OC use for women (OR = 4.67; *P* < .0001) were highly significant risk factors. By contrast, the social status was no longer significant. 

Table 4 examines the probability of vitamin C and *β*-carotene deficiency (i.e., lower than the critical values) with respect to an increasing number of risk factors. Healthy habits consisting in no smoking, practising a regular physical activity, and eating at least two fruits per day resulted in a weak probability (less than 5%) to have plasma vitamin C below 6 mg/L or 34.2 *μ*M. By contrast, poor healthy living habits (smoking, no physical activity, nonconsumption of fruits, and overweight) significantly increased this probability up to 46.2% for men against 33.5% for women. When accounting for unemployment, these probabilities raised to 66.3% for men and 44.3% for women, respectively. It should also be noted that men were always associated with higher probabilities of vitamin C levels below 6 mg/L than women (except those of social class IV and Va) smoking, practising no physical activity, and eating none fruits regardless of the combination of lifestyle behaviours. For *β*-carotene, healthy habits (nonsmoking, practising sport, and eating two or one fruits per day) resulted in 13.7% and 29.7% of chance to get critical level in *β*-carotene for women and men, respectively. Smoking by itself significantly increased this probability but in a higher extent in men (56.1%) than in women (32.4%). The combination of smoking, absence of regular physical activity, and any intake of fruits contributed to a high probability in men (73.2%) and to a less extent in women (50.5%). The addition of overweight resulted in an increase of about 20% in women (69.9%) and of only 13% in men (86.1%). Finally, the worst situation (91.6%) was observed for women by adding the intake of oral contraceptive pills. It should be noted that for both genders, all probabilities were largely higher than those derived for vitamin C risk of deficiency.

## 4. Discussion

### 4.1. Reference Values

The reference values for vitamin C established by our group are comprised between 6.2 mg/L (35.3 *μ*M) and 18.8 mg/L (107.1 *μ*M). This is in agreement with other studies performed in different European populations [20, 35]. In the work of Rutkowski and Grzegorczyk [36], ten ranges of concentrations given by medical handbooks and textbooks have been compared in detail with 15 parallel ranges taken from scientific papers, paying attention to their significant discrepancies. Based on source values and basic statistical calculations, a reliable mean range of vitamin C “normal concentrations" in blood plasma has been obtained: 6.3–14 mg/L (36.1–79.4 *μ*M). According to Le Grusse and Watier [37], the critical range of accepted marginal vitamin C deficiency was between 3.5 and 6.2 mg/L (20 *μ*M–35 *μ*M). For *β*-carotene, we found the following normal range 0.05–0.62 mg/L (0.09–1.15 *μ*M) which is in good agreement with the study of Olmedilla et al. performed in five Western European populations [35]. 

#### 4.1.1. Mean Plasma Antioxidant Concentration

With respect to gender, our mean values in antioxidants were in perfect agreement with those found in the reference French SUVIMAX study (vitamin C: men: 8.8 ± 4.0 mg/L; women: 10.6 ± 5.5 mg/L; *P* < .0001; *β*-carotene: men: 0.22 ± 0.16 mg/L; women: 0.31 ± 0.20 mg/L; *P* < .0001) [20, 38]. Fruits and to a less extent vegetables are the primary dietary sources of both antioxidants. As confirmed in Table 1, it is recognized that women have dietary intakes richer in vitamin C and *β*-carotene than men due to a higher intake of fruit and vegetables. As expected, our data clearly indicate that smoking was associated with a significant decrease of the mean plasma vitamin C (19%) and *β*-carotene (39%) after adjustment for gender and all demographic variables described in Table 1. Our observations were in good agreement with other reports [26, 27] and, more particularly, the SUVIMAX study performed on 3128 French men and women aged 35–60 years [38]. Several explanations can be proposed for explaining this observation. Cigarette smoke contains a large number of free radicals species able to induce an oxidative stress on both the respiratory and circulatory systems with as consequence greater antioxidant depletion. Smoking also contributes to a significant reduced intake of fruits rich in both antioxidants. After adjustment for all covariates, we also evidenced that a regular physical activity contributes to improve the vitamin C and *β*-carotene levels. A simple explanation could be given by the fact that active people eat more fruits than inactive ones. The recent Man study among 455 Latino and African American men in the U.S. Southeast showing a significant association between fruit and vegetable consumption and physical activity (*P* < .001) confirms our findings [39]. We also evidenced a positive relationship between the frequency of fruit and the mean plasma level of vitamin C and *β*-carotene. When compared to high consumers, people eating any fruit were characterized by plasma concentrations of both antioxidants which are extremely closed to the normal inferior value. By contrast, we were unable to evidence an association between antioxidant biomarkers and the consumption of vegetables. The poor intake of vegetables in the ELAN cohort could partially explain this observation. Moreover, we also evidenced that only the intake of carrot and tomato among vegetables may significantly influence to a higher extent the plasma level of *β*-carotene whilst a larger range of fruits were able to do it. It could be also possible that *α*-carotene is a better predictor than *β*-carotene as suggested in the European Prospective Investigation into Cancer and Nutrition (EPIC study) performed on a stratified random subsample of 3089 men and women [40]. Ideal BMI is the range of 20–25 kg/m^*2*^ while a BMI of over 25 kg/m^*2*^ and 30 kg/m^*2*^ is, respectively associated with overweight and obesity. As described earlier [41, 42], we confirmed that a BMI > 25 kg/m^2^ resulted in a significant decrease by 31% of the plasma *β*-carotene level. The use of oral contraceptives had a deep negative impact on the plasma level of *β*-carotene [31]. It has been speculated that estrogens induce an activation of the retinol binding protein [43], hence possibly increasing the conversion of *β*-carotene into retinol. We have also shown that women taking oral contraceptives had higher oxidative damages to lipids than the others [31]. It could be assumed that part of the antioxidant defences were more solicited in women using oral contraception to limit deleterious damages. Neither blood pressure nor intestinal disorders (which could explain a decrease in antioxidants due to possible malabsorption) or environmental parameters had an influence on the mean plasma level of both antioxidants.

#### 4.1.2. Cutoff Values

Based on large-scale epidemiological studies, it is now accepted that an alteration of antioxidant defences was significantly implicated in the development of several pathologies. However, if some variations of the mean plasma values may be evidenced with respect to lifestyle behaviours in all studies, no indication was ever given about the biological interpretation of these variations. Therefore, the establishment of reference or usual values for antioxidants markers such vitamin C and *β*-carotene is needed to detect abnormalities. As explained above, determination of normal ranges for both antioxidants has been achieved on a separate population of 128 healthy persons as earlier published [31–33]. Based on our reference values, we were able to detect that 16.4% of the whole ELAN population had non optimal plasma concentration in vitamin C (<6 mg/L or 34.2 *μ*M) against 46.7% for *β*-carotene (<0.22 mg/L or 0.4 *μ*M). 

At the light of the following but not exhaustive studies, the evidence of such abnormalities could therefore be of primordial interest as a preventive tool for health [44, 45]. Recently, Langlois et al. [46] proposed that plasma vitamin C should be considered as a predictor of cardiovascular disease in addition to being a classical nutritional biomarker. Based on a large number of studies including the famous Monica study [14, 18] performed on 14 European populations, plasma cutoff levels (4.4–7.0 mg/L or 25–40 *μ*M) in vitamin C have been proposed, above which the risk for apparent cardiovascular events should decrease. In haemodialysis patients, the cutoff value of 5.66 mg/L (32 *μ*M) was predictive of the appearance of adverse cardiovascular outcomes [47]. In patients with peripheral arterial disease, the cutoff value of 4.9 mg/L (28 *μ*M) was associated with increased levels of inflammation parameters [48]. Recently, Myint et al. [49] described in the European Prospective Investigation into Cancer (EPIC)-Norfolk population that the relative risks for risk of stroke diminished inversely to the quartile of plasma vitamin C concentration as follows: 1.0 (<41 *μ*M or 7.27 mg/L), 0.83 (41–53 *μ*M or 7.27–9.32 mg/L), 0.63 (54–65 *μ*M or 9.5 or 11.44 mg/L), and 0.57 (>66 *μ*M or 11.6 mg/L). In the same study, it was interesting to highlight that there was a continuous relation with mortality through the whole distribution of ascorbic acid concentrations. When compared to 3.66 mg/L, each 3.52 mg/L (20 *μ*M) rise in plasma ascorbic acid concentration was associated with about a 20% reduction in risk of all-cause mortality (*P* < .0001), regardless of age, systolic blood pressure, blood cholesterol, cigarette smoking habit, diabetes, and supplement use. For *β*-carotene, Gey established that a plasma value below 0.22 mg/L (0.4 *μ*M) was associated with an increased risk of developing cardiovascular diseases and cancer [18]. 

Among the ELAN population, 16.4% of the subjects presented a plasma vitamin C below the cutoff value of 6 mg/L (34.2 *μ*M) and 46.7% a concentration in *β*-carotene below the critical point of 0.22 mg/L (0.4 *μ*M).

#### 4.1.3. Predictive Model

The availability of a statistical model for predicting the probability of getting a plasma value below the cutoff values of 6 mg/L (34.2 *μ*M) for vitamin C and 0.22 mg/L (0.4 *μ*M) for *β*-carotene could be of interest for health prevention. Cumulative probabilities were therefore presented instead of odd ratios to stress on the cumulative effect of the risk factors on low levels in both antioxidants. After adjustment for all covariates between them, Table 3 clearly indicates that using such values rather than mean plasma value afforded better indications about the relationship between antioxidant biomarkers and lifestyle behaviours. A good base of healthy life could be nonsmoking, regular physical activity and eating more than 3 fruits/day. This resulted in a small probability to have inadequate plasma level of vitamin C. Smoking and nonphysical activity significantly but moderately contributed to increase this probability for vitamin C for both genders. In contrast, the nonconsumption of fruits added to the parameters above produced a dramatic increase which was more pronounced in men (42.1%) than in women (30% only for those belonging to social classes I, II, IIIa, and IIIb). This suggests that the frequncy of fruit intake appears to be one of the most important regulators of the plasma concentration of vitamin C [50, 51] with a more pronounced effect in men (65.3%) having partial or no working activity. Economical difficulties to buy fruits linked to a precarious social status can explain this last observation. As observed with the mean plasma values, no effect of BMI, intestinal disorders, and environment could be evidenced on the probability to get low plasma level in vitamin C. 

With respect to *β*-carotene, it was quite interesting to note that despite a healthy way of life there was a significant risk (29.7% in men and 13.7% in women) to get a value below 0.22 mg/L (0.4 *μ*M). Malabsorption of this antioxidant could be a rationale explanation although we did not find any influence of intestinal disorders on the plasma level of *β*-carotene. When compared to basal level and according to the different steps described in table, smoking contributes to a mean increase of 27% for men and 18% for women of getting critical plasma value. This is not surprising since the negative impact of smoking on antioxidant is well known [20]. Lack of physical activity was associated with a further but moderate increase of around 9%. The lack of fruits consumption resulted to a subsequent raise of 8%. This was less pronounced than those observed for vitamin C confirming that the latter is a better biomarker than *β*-carotene of fruits intake. In contrast to vitamin C, two additional parameters, BMI > 25 kg/m^2^ and oral contraceptives in women, significantly contributed to finally reach a high probability (86.1% for men and 91.6% for women) to detect nonoptimal plasma value in *β*-carotene.

#### 4.1.4. Limitations of the Study

Although representative of the Province of Liège, the study sample may not be considered as a national probability sample of the Belgian population. Moreover, we only focused our attention on people in the age range of 40–60 years since we considered that lifestyle behaviours were well anchored in this population. It is clear that ageing (>60 years) could also contribute to decrease the plasma level in antioxidants so that our observations in the ELAN population could be modified if taking this parameter in account. The measurement of exposure to cigarette smoking has some limitations. We only distinguished nonsmokers from both past and current smokers, this last one category being, however, in a large majority in the ELAN population. Further, no question has been addressed with respect to environmental tobacco smoker which could possibly have a negative influence on plasma antioxidants. During our study, we also met some difficulties to integrate data about alcohol consumption due to a large underestimation of intake made by participants. As this parameter has been shown to reduce the level of plasma *β*-carotene, it could partially explain the relatively high probability of getting a value below 0.22 mg/L (0.4 *μ*M) even when observing optimal lifestyle behaviours (no smoking, physical activity and eating more than ≥2 days a day). Except for oral contraceptive, the influence of other drugs intake was not taken into consideration.

## 5. Conclusions

To the best of our knowledge, the present study is the first one to address the relationship between plasma antioxidants and lifestyle behaviours in a Belgian population. We have demonstrated that smoking regular physical activity and eating fruits were directly associated with the modulation of the mean plasma concentration of both vitamin C and *β*-carotene. However, such variations, if well described in the literature, always remained within the normal or usual range of concentration. By using cutoff values associated with increased risk of developing cardiovascular diseases and cancer (vitamin C < 6 mg/L or 34.2 *μ*M and *β*-carotene <0.22 mg/L or 0.4 *μ*M), we described how lifestyle factors alone or associated contribute to lower plasma concentrations. The present approach should help general practitioners in disease promotion. However, as vitamin C and *β*-carotene are unstable constituents when not protected against air and light, a rigorous preanalytical sample handling and treatment (immediate centrifugation, plasma precipitation and keeping the sample at −80°C until analysis) is required to interpret the data correctly. This is unfortunately rarely achieved in most laboratories [33].

##  Authors' Contribution

The ELAN (“Etude Liégeoise sur les ANtioxydants”) study was conducted from March through July 2006 as a joint project between the University of Liège, the University Hospital of Liège, and the local health services of the Province of Liège (Belgium). Professor JO Defraigne and Dr. Sc J Pincemail (CREDEC and Dept of Cardiovascular Surgery) were the main coordinators of the ELAN study. They collected all data and wrote the manuscript in the present form. Professor C Charlier and Professor JP Chapelle (Laboratories of Clinical Biology) allowed the analysis of *β*-carotene while vitamin C determination was performed by Mr JP Cheramy-Bien (CREDEC). Professor D Giet and Dr. G Collette (Dept of General Medicine) allowed the recruitment of all general practitioners around the Province of Liège, Belgium. Professor A Albert and Dr. Sc S Vanbelle (Dpt of Medical Informatics and Biostatistics) were involved in the statistical analysis of all data. All investigators critically revised the manuscript for the intellectual content and gave their final approval of the version to be published.

## Figures and Tables

**Table 1:** Gender specific demographic, biometric, medical and dietary characteristics of the ELAN population. See text for description of social class.

Variable	Women	Men	*P* value
	*n* = 548	*n* = 349	
Age (years)	50.1 ± 5.8	50.9 ± 6.0	.057
Smoking			
No	402 (73)	262 (75)	.55
Yes	145 (27)	86 (25)	
Height (m)	1.64 ± 0.07	1.77 ± 0.07	<.0001
Weight (kg)	67.2 ± 13.4	83.6 ± 13.3	<.0001
BMI (kg/m²)	25.0 ± 4.7	26.8 ± 3.8	<.0001
Systolic blood pressure (mmHg)	123 ± 14	128 ± 13	<.0001
Diastolic blood pressure (mmHg)	76 ± 8.8	79 ± 9.6	<.0001
Intestinal disorders			
No	429 (79)	301 (86)	.0039
Yes	117 (21)	48 (14)	
Physical activity			
No	339 (62)	202 (58)	.19
Yes	205 (38)	147 (42)	
Living environment			
Rural	195 (36)	126 (36)	.69
Semi urban	152 (28)	104 (30)	
Urban	201 (37)	119 (34)	
Dietary intakes (g/day)			
Total vitamin C	142 ± 68.7	118 ± 70.1	<.0001
Vitamin C (fruit)	96.6 ± 58.8	75.1 ± 58.8	<.0001
Vitamin C (vegetables)	47.3 ± 27.8	43.3 ± 25.7	<.0001
Total *β*-carotene	4.9 ± 3.1	4.0 ± 2.6	<.0001
Total *β*-carotene (fruit)	1.8 ± 2.3	1.2 ± 1.9	<.0001
Total *β*-carotene (vegetables)	3.1 ± 1.7	2.8 ± 1.6	.045
Social class			
I	29 (5.5)	51 (15.0)	<.0001
II	1 (0.2)	14 (4.1)	
IIIa	9 (1.7)	34 (10.0)	
IIIb	245 (46.1)	115 (33.9)	
IV	59 (11.1)	66 (19.5)	
Va	25 (5.1)	38 (11.2)	
Vb	162 (30.5)	21 (6.2)	

**Table 2:** Vitamin C and *β*-carotene plasma concentrations according to subject's characteristics. Data are expressed as mean ± SD.

Characteristics	Vitamin C (mg/L)	*P* value	*β*-carotene (mg/L)	*P* value
Gender				
Men	9.0 ± 3.4	<.0001	0.24 ± 0.18	<.0001
Women	10.5 ± 4.1		0.35 ± 0.28	
Smoking				
No	10.4 ± 3.7	<.0001	0.34 ± 0.27	<.0001
Yes	8.5 ± 4.1		0.21 ± 0.15	
BMI				
<25 kg/m²	10.3 ± 3.9	.0026	0.36 ± 0.30	<.0001
>25 kg/m²	9.5 ± 3.9		0.25 ± 0.19	
Intestinal disorders				
No	9.8 ± 3.9	.48	0.31 ± 0.26	.40
Yes	10.1 ± 3.8		0.29 ± 0.21	
Physical activity				
No	9.5 ± 4.2	<.0005	0.28 ± 0.24	<.0001
Yes	10.4 ± 3.3		0.35 ± 0.26	
Living environmental				
Rural	10.1 ± 3.6	.31	0.30 ± 0.22	.40
Semi rural	9.6 ± 3.6		0.31 ± 0.29	
Urban	9.8 ± 3.9		0.32 ± 0.26	
Oral contraceptives				
No	9.8 ± 3.9	.26	0.36 ± 0.27	.0020
Yes	10.8 ± 4.2		0.22 ± 0.18	
Fruit consumption				
None	8.4 ± 4.2	<.0001	0.23 ± 0.2	.0001
1-2 fruits/day	9.7 ± 3.8		0.30 ± 0.24	
2-3/fruits/day	11.2 ± 3.9		0.35 ± 0.24	
>3 fruits/day	11.5 ± 2.9		0.42 ± 0.37	
Social class				
I	9.9 ± 3.2	.12	0.35 ± 0.24	.0006
II	9.7 ± 3.2		0.28 ± 0.27	
IIIa	9.4 ± 3.5		0.26 ± 0.23	
IIIb	10.4 ± 3.5		0.34 ± 0.28	
IV	9.5 ± 4.6		0.26 ± 0.24	
Va	9.5 ± 3.2		0.22 ± 0.13	
Vb	9.5 ± 4.6		0.29 ± 0.21	

**Table 3:** Risk factors related to vitamin C and *β*-carotene deficiency. Results are expressed as OR with 95% confidence intervals.

Risk factor	Vitamin C OR (95% CI)	*β*-carotene OR (95% CI)
Gender (Male)	1.70 (1.13–2.57)	2.67 (1.95–3.66)
Smoking (Yes)	2.84 (1.90–4.25)	3.03 (2.13–4.32)
Overweight^(a)^ (Yes)	1.18 (0.79–1.77)^(b)^	2.28 (1.68–3.10)
Physical activity (No)	2.05 (1.31–3.20)	1.51 (1.11–2.05)
Fruits (<2/day) (Yes)	2.96 (1.64–5.34)	1.41 (1.01–1.98)
OC (Yes)	1.24 (0.54–2.86)^(b)^	4.67 (2.52–8.67)
Unemployed (Yes)	3.31 (1.30–8.45)	1.13 (0.61–2.11)^(b)^

^(a)^BMI ≥ 25 kg/m².

^(b)^Not significant.

**Table 4:** Probability of developing vitamin C deficiency and *β*-carotene deficiency according various risk factors combinations (from no risk factor present to all risk factors present) in men and women. For vitamin C, results in parentheses indicate probability for employed and unemployed subjects.

					Probability of getting a plasma value of vitamin C below 6 mg/L^(b) ^		probability of getting a plasma value of *β*-carotene below 0.22 mg/L	
Smoking	Physical activity	Fruits intake (≥2/day)	Overweight^(a)^	OC use	Men	Women	Men	Women
No	Yes	Yes	No	No	4.0 (3.5–10.4)	2.4 (1.5–4.5)	29.7	13.7
Yes	Yes	Yes	No	No	10.7 (9.2–24.3)	6.6 (3.9–11.5)	56.1	32.4
Yes	No	Yes	No	No	19.7 (16.9–39.3)	12.6 (7.6–20.7)	65.9	42.0
Yes	No	No	No	No	42.1 (37.1–65.3)	30.0 (19.3–43.2)	73.2	50.5
Yes	No	No	Yes	No	46.2 (38.2–66.3)	33.5 (20.0–44.3)	86.1	69.9
Yes	No	No	Yes	Yes	(NA)	38.5 (24.3–50.4)	(NA)	91.6

^(a) ^BMI ≥ 25   kg/m².

^(b) ^Numbers in parentheses indicated probability of vitamin C deficiency when subject being employed or unemployed.
